# Exogenous Nitric Oxide Induces Pathogenicity of *Alternaria alternata* on Huangguan Pear Fruit by Regulating Reactive Oxygen Species Metabolism and Cell Wall Modification

**DOI:** 10.3390/jof10100726

**Published:** 2024-10-19

**Authors:** Di Wang, Haijue Zhang, Lingkui Meng, Xinyu Tan, Rong Liu, Qingchao Gao, Yan Wu, Yuhan Zhu, Xueyan Ren, Yongcai Li, Qingjun Kong

**Affiliations:** 1 Xi’an Key Laboratory of Characteristic Fruit Storage and Preservation, Shaanxi Engineering Laboratory of Food Green Processing and Safety Control, College of Food Engineering and Nutritional Science, Shaanxi Normal University, Xi’an 710119, China; wangdi237@snnu.edu.cn (D.W.); zhanghaijue@snnu.edu.cn (H.Z.); mlk@snnu.edu.cn (L.M.); tt707294@snnu.edu.cn (X.T.); xieyao-bingganbuqia@snnu.edu.cn (R.L.); gqc199411@163.com (Q.G.); rxy104@snnu.edu.cn (X.R.); 2 College of Food Science and Engineering, Gansu Agricultural University, Lanzhou 730070, China

**Keywords:** nitric oxide, *Alternaria alternata*, pathogenicity, ROS metabolism, cell degradation, pear fruit

## Abstract

Black spot caused by *Alternaria alternata* is one of the most common postharvest diseases in fruit and vegetables. A comprehensive investigation into its pathogenicity mechanism is imperative in order to propose a targeted and effective control strategy. The effect of nitric oxide (NO) on the pathogenicity of *A. alternata* and its underlying mechanism was studied. The results showed that treatment with 0.5 mM L^−1^ of sodium nitroprusside (SNP) (NO donor) increased the lesion diameter of *A. alternata* in vivo and in vitro, which was 22.8% and 13.2% higher than that of the control, respectively. Exogenous NO treatment also induced endogenous NO accumulation by activating nitric oxide synthase (NOS). In addition, NO triggered an increase in reactive oxygen species (ROS) levels. NO enhanced activities and gene expression levels of NADPH oxidase (NOX), superoxide dismutase (SOD), catalase (CAT), ascorbate peroxidase (APX), glutathione peroxidase (GPX), and glutathione reductase (GR). Moreover, NO stimulated cell wall degrading enzymes by activating the corresponding gene expression in vivo and in vitro. These results suggested that exogenous NO promoted the pathogenicity of *A. alternata* by inducing ROS accumulation and activating antioxidants and cell wall degrading enzymes. The present results could establish a theoretical foundation for the targeted control of the black spot disease in pear fruit.

## 1. Introduction

Fresh fruits and vegetables are highly susceptible to infection by pathogenic fungi during postharvest storage and transportation, resulting in decay that seriously affects their quality [1]. *Alternaria alternata* is one of the most common rot-causing fungi of fruits and vegetables, causing black spot disease on a variety of produce such as apples, pears, grapes, and peaches, leading to significant postharvest losses [2]. Additionally, the toxins produced by *A. alternata*, including tenuazonic acid (TeA), alternariol (AOH), and alternariol monomethyl ether (AME), are harmful to human health [2]. To address these issues, many studies have focused on inhibiting *A. alternata*, but few have investigated its pathogenicity during infection. Therefore, it is necessary to study the pathogenic mechanisms of *A. alternata* to provide a theoretical basis for precise control strategies.

Chemical control is the use of chemicals to control plant diseases and is one of the most widely used and effective methods. However, the long-term use of chemicals can lead to the development of resistance in pathogens, which reduces the effectiveness of chemical control. In addition, the pollution of the environment and harm to the human body caused by chemical agents has become a major concern of people today. Biological control is attracting more and more attention due to its advantages of safety and harmlessness to the human body and environment [1]. Commonly used biocontrol agents are mainly antagonistic bacteria or fungus and their by-products, natural compounds extracted from plants and animals or microorganisms [3,4]. However, environmental conditions such as temperature, humidity, rainfall, etc., can affect the colonization and growth of antagonistic bacteria on the surface of fruits and vegetables, thus reducing the effectiveness of bacterial inhibition. In addition, the high cost of producing antagonistic bacteria of fungus and natural antimicrobial substances limits their use in postharvest fruit and vegetable preservation [3].

Nitric oxide (NO) is an important signaling molecule involved in various key signaling pathways and regulates a variety of physiological processes. In recent years, the application of exogenous NO as an efficient and environmentally friendly gaseous preservative for postharvest disease control in fruits and vegetables has received increasing attention. Some studies have shown that high concentrations of NO can reduce postharvest diseases by affecting the growth and development of pathogenic bacteria and improving the resistance of fruits and vegetables (literature). For example, exogenous NO can significantly control postharvest diseases in horticultural products such as grape [5], wheat [6], onion [7], and potato [8].

NO is mainly derived from nitric-oxide-synthase-like (NOS-like) enzymes in fungi, which convert arginine to citrulline, resulting in NO production [9]. NO is related to several signaling pathways in fungi, including cyclic guanosine monophosphate (cGMP), mitogen-activated protein kinase (MAPK), and light signaling pathways. NO can activate soluble guanylate cyclase (sGC) activity, which catalyzes the conversion of guanosine triphosphate (GTP) to cGMP. Subsequently, cGMP, combined with NO, functions through the NO-cGMP signaling pathway [10]. It has been reported that low levels of NO affect spore production in *Puccinia striiformis* [6], growth and development in *Stemphylium eturmiunum* [7], and appressorium formation in the rice blast fungus [8]. Additionally, NO has been found to regulate spore formation and germination in *B. emersonii* and *Schizosaccharomyces pombe* by mediating the cGMP signaling pathway [11]. Conversely, high levels of NO can damage cellular structures and inhibit fungal growth and development. To date, the inhibitory effects of elevated NO have been reported in *Fusarium sulphureum* [8], *Aspergillus flavus* [12], *Aspergillus niger* [13], and *Penicillium italicum* [13]. However, studies on the pathogenicity of *A. alternata* regulated by low levels of NO and its mechanisms are scarce.

During plant infections, both pathogens and plants experience a burst of reactive oxygen species (ROS) [14]. In fungi, hydrogen peroxide (H_2_O_2_) and superoxide (O_2_·*^−^*) are common ROS, mainly produced by reduced nicotinamide adenine dinucleotide phosphate oxidase (NADPH oxidase, NOX). NOX generates ROS by transferring electrons from NADPH to molecular oxygen [14]. As a key signaling molecule, ROS has a dual role in plants, animals, and microorganisms. Suitable levels of ROS act as a second messenger in regulating various physiological and metabolic activities. However, excessive ROS cause oxidative damage to proteins, DNA, and lipids [14]. The inhibitory effects of high levels of ROS have been demonstrated in *Magnaporthe grisea* [3], *Aspergillus flavus* [15], *Aspergillus ochraceus* [15], *Fusarium oxysporum* [16], and *Botrytis cinerea* [16]. Meanwhile, appropriate concentrations of ROS are important for the physiological activities of pathogens, including mycelial growth, conidial differentiation, and formation of substrate infestation structures [17]. Low levels of ROS are necessary for appressorium formation during infection in *Puccinia triticina* [17] and the rice blast fungus [18], essential for developing the infection process in pathogens. Accordingly, ROS production or scavenging at specific stages during pathogen infection is critical [19]. The reactive oxygen scavenging system of pathogens, including antioxidant enzymes such as superoxide dismutase (SOD), catalase (CAT), ascorbate peroxidase (APX), glutathione peroxidase (GPX), and glutathione reductase (GR), is activated during infection to reduce excess ROS in pathogen intracellular and plant tissues, ultimately preventing oxidative damage to cells [19]. Zhang et al. [20] found that knocking out the ROS-producing gene NOXR decreased levels of O_2_·*^−^* and H_2_O_2_ in the mycelium, leading to reduced SOD and CAT activities. Studies have reported that exogenous H_2_O_2_ induced endogenous ROS accumulation in *A. alternata*, accompanied by increased SOD, CAT, and APX activities in response to oxidative stress [21]. Antioxidant genes were upregulated after the successful colonization of plant roots by *Arbuscular Mycorrhizae* [22]. Moreover, the loss of SOD1 activity in *Oidiodendron maius* increased the fungus’ sensitivity to ROS [23]. These studies highlight the importance of the ROS scavenging system for pathogen infection and pathogenicity.

Plant cell walls are composed of cellulose, pectin, and hemicellulose, which act as protective barriers against pathogen infection [24]. In response, pathogens synthesize cell-wall-degrading enzymes (CWDEs) during infection, damaging plant tissues and accelerating pathogen infection [24]. Pathogens secrete different types of CWDEs, and it is generally believed that pathogenicity is closely related to CWDE activity [24]. Common CWDEs in fungi include cellulase (Cx), β-1,3-glucanase, polygalacturonase (PG), pectin methylesterase (PME), pectin methylgalacturonase (PMG), polygalacturonate trans-eliminating enzyme (PGTE), and pectin methyl trans-eliminating enzyme (PMTE) [25]. PMG, PG, Cx, and β-glucosidase secretion have been detected in *Rhizoctonia solani* in vitro and in tobacco tissues, promoting the development of the infection process [26]. *Fusarium equiseti* was found to secrete PG and cause fusarium wilt when infecting pitaya fruit, and PG gene knockout mutants showed reduced pathogenicity [27]. Therefore, CWDEs secreted by the pathogen might play a pathogenic role as virulence factors during host plant infection.

Most of the research has focused on inhibiting *A. alternata* and inducing resistance in the host via NO. Few studies have investigated the effect of NO on the pathogenicity of *A. alternata*. To further understand the role of exogenous NO, different concentrations of SNP (NO donor) were applied to investigate the effect of exogenous NO on endogenous NO, ROS, and cell wall degradation pathways, clarifying the potential regulatory mechanisms of NO in the pathogenicity of *A. alternata*.

## 2. Materials and Methods

### 2.1. Chemicals and Reagents

Sodium nitroprusside (SNP) (99%, AR) was purchased from Aladdin Reagent (Shanghai, China). Standards of cGMP (≥98%), ergosterol (≥98%), and high efficiency liquid chromatography (HPLC) grade methanol (≥99%) were purchased from Sigma Chemical Co. (St Louis, MO, USA). All other reagents were of analytical grade and were purchased from Shanghai Macklin Biochemical Co., Ltd. (Shanghai, China).

### 2.2. Pear Fruit and Pathogen; Treatment and Storage

*A. alternata* was obtained from the College of Food Science and Engineering, Gansu Agricultural University, China. *A. alternata* was subcultured on potato dextrose agar (PDA) at 25 °C for 7 days, and the conidial suspension (10^6^ spores × 10^−3^ L^−1^) was prepared for inoculation.

‘Huangguan’ pear fruits (*Pyrus bretschneideri* Rehd.) (firmness of 78 *n* ± 2 *n*, total soluble solids content of 11.4 ± 2.6%, titrable acid of 0.13 ± 0.06%) with uniform size and without injury and disease were selected from a market in Xi’an City, Shaanxi Province, China, and immediately transported to the laboratory. The fruits were immersed in sodium hypochlorite and washed with sterile water. After air drying, the fruits were used for inoculation.

### 2.3. Spore Germination Assay

The spore germination assay was conducted based on the method of Li et al. [2], with minor modifications. At length, sodium nitroprusside (SNP) was added to sterile water to form a solution at concentrations of 0.25, 0.5, 0.75, and 1 mM L^−1^, and sterile water treatment was used as a control. The serial gradient SNP solution and sterile water were added to the surface of sterile water agar cakes (8 mm in diameter), and then 10 μL of spore suspension (1 × 10^6^ spores mL^−1^) continued to be dropped on the surface of the sterile water agar, respectively. Petri dishes containing spores were incubated at room temperature for 12 h. The rate of spore germination was determined at every 2 h at 25 °C under a light microscope. The spore germination rate was determined every 2 h under a light microscope (Olympus, Tokyo, Japan). Approximately 200 spores were selected and counted using a hemocyte counting plate to calculate the spore germination rate. Three replicates were used for each group.

### 2.4. In Vitro Mycelial Growth and In Vivo Pathogenicy Assessment of A. alternata

The assessment of in vitro mycelial growth and in vivo pathogenicity was based on the method of Li et al. [2], with minor modifications. *A. alternaria* was cultured on potato dextrose agar (PDA) for seven days, and the spore suspension (10^6^ spores × 10^−3^ L^−1^) was prepared for inoculation. A total of 300 pear fruits were selected and randomly divided into two groups of 150 each (control group and elicitor-treated groups), including 3 replicates. The pear fruits were surface sterilized with 75% alcohol, air dried, and then uniformly punched on the surface of the tubers with a perforator (4 mm in diameter). The holes were inoculated with 20 μL of spore suspension and 5 μL of different concentrations of SNP solution (0.25, 0.5, 0.75, 1 mM L^−1^) and inoculated with 20 μL of spore suspension and 5 μL of sterile water as a control, then dried and stored at 25 (±2 °C). Wounded inoculated fruits were stored at 22 °C, and the diameter of black spot lesions was determined by the criss-cross method after 16 days.

**Figure 1:**
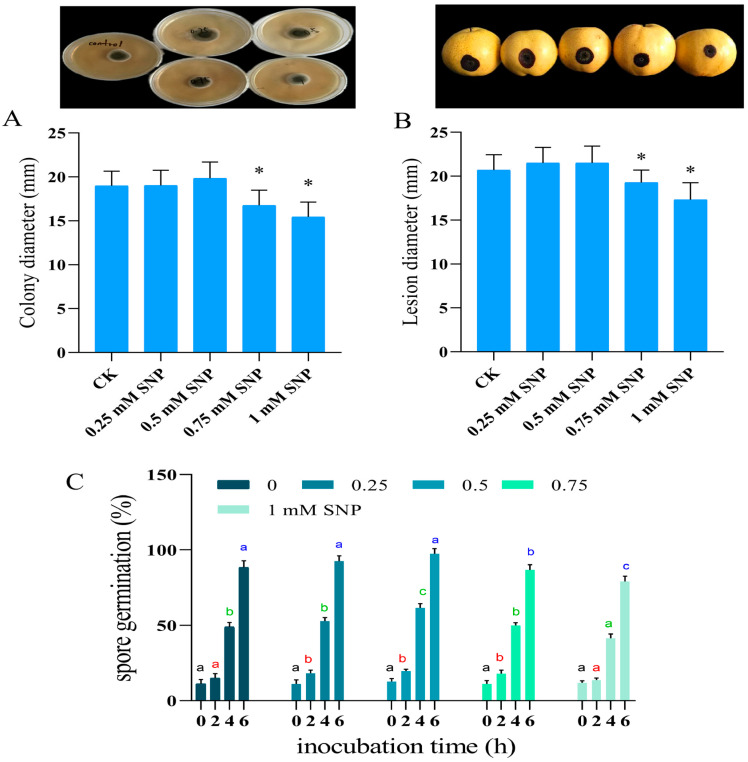
Effect of SNP on colony diameter in vitro (**A**), diameter of black spot disease in vivo (**B**), and spore germination rate of pear fruit (**C**). Values are presented as means ± SD (*n* = 10). The different letters and * indicate significant differences (*p* < 0.05).

Agar discs containing *A. alternata* mycelium were taken using a punch and inoculated in the center of PDA plates. Sterile water and 0.25, 0.5, 0.75, and 1 mM concentrations of SNP solution were then added to the surface of the medium, respectively. The culture was then incubated at 22 °C for 5 d. Mycelial growth was observed and the diameter of the *A. alternata* in vitro was determined after 5 d using the criss-cross method. A further 0.5 mM NO was selected as the best from preliminary experiments (according to the data shown in Figure 1 and Figure S1).

### 2.5. Measurement of Endogenous NO and NOS Activity

Endogenous NO was determined using the NO assay kit (S0021S, Beyotime Biotechnology, Shanghai, China) according to the manufacturer’s instructions. The measured NO content of pear fruit samples was expressed as μmol g^−1^ protein. NOS activity was determined using a nitric oxide synthase (NOS) type assay kit (S0025, Beyotime Biotechnology, Shanghai, China) according to the manufacturer’s instructions. The measured NOS activity of *A. alternata* samples was expressed as U mg^−1^ protein.

### 2.6. Measurement of cGMP Content

The cGMP content was detected according to the method of Soleimani Aghdam et al. [28], with slight modification. Frozen mycelium samples (1 g) were extracted with PBS buffer (pH 7.2) at room temperature by ultrasonic extraction for 30 min and then centrifuged at 12,000× *g* at 4 °C for 15 min. The supernatant was collected for measurement. The cGMP content was determined using an Ultra Performance Liquid Chromatography (UPLC) system (Agilent Technologies Co, Santa Clara, CA, USA) equipped with a C_18_ column (ACQUITY UPLCROligonucleotide BEH C_18_ column, 2.1 mm × 100 mm, 1.7 μm) (Waters Corporation, Milford, MA, USA). The Version B.10.0 software (Agilent, Lake Forest, CA, USA) was used to analyze the experimental data. The mobile phase was composed of a solution containing 0.02 moL L^−1^ of potassium dihydrogen phosphate in methanol (pH 2.38) at a ratio of 4:96. The injection volume was set at 3 μL, and the flow rate of 0.25 mL min^−1^ and column temperature of 30 °C were maintained. The UV detection wavelength was set at 254 nm, and the cGMP content was expressed as μmol g^−1^ on a dry weight (DW) basis.

### 2.7. Detection of Endogenous NO and ROS by 3-Amino, 4-Aminomethyl-2′, 7′-difluorescein diacetate (DAF-FM) and 2, 7-Dichlorodi-hydrofluorescein diacetate (DCHF-DA) Fluorescent Staining

**Figure 2:**
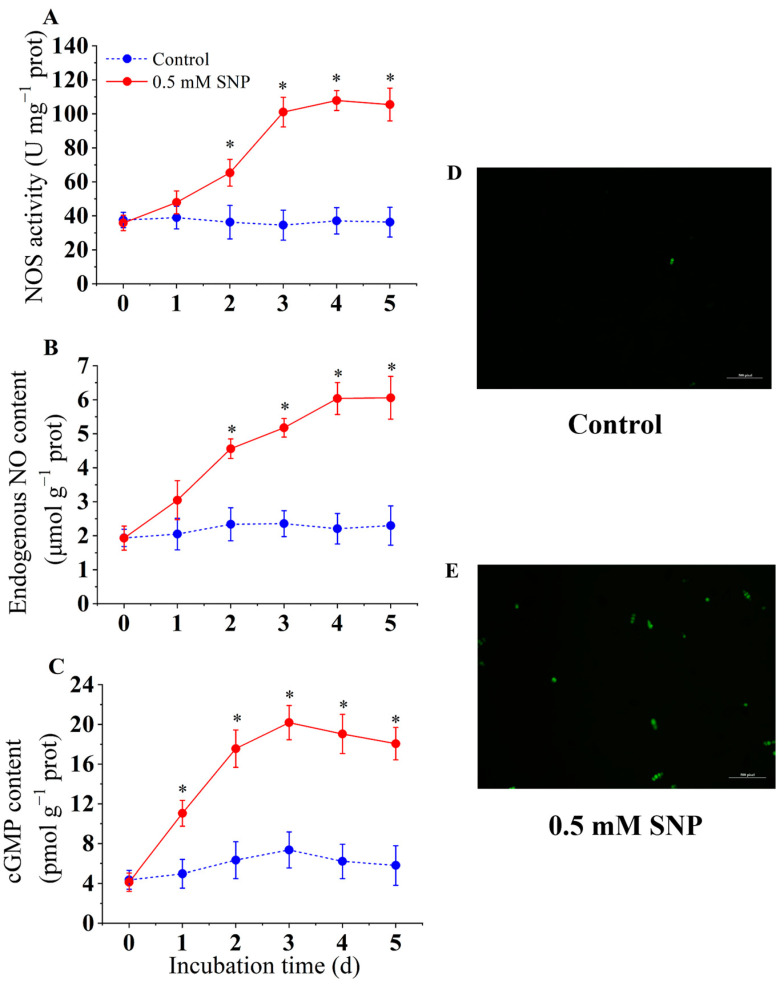
Effect of SNP on NOS activity (**A**), endogenous NO content (**B**), cGMP content (**C**), NO fluorescence (**D**,**E**) of *A. alternata*. Values are presented as means ± SD (*n* = 10). The * indicates significant differences (*p* < 0.05).

**Figure 3:**
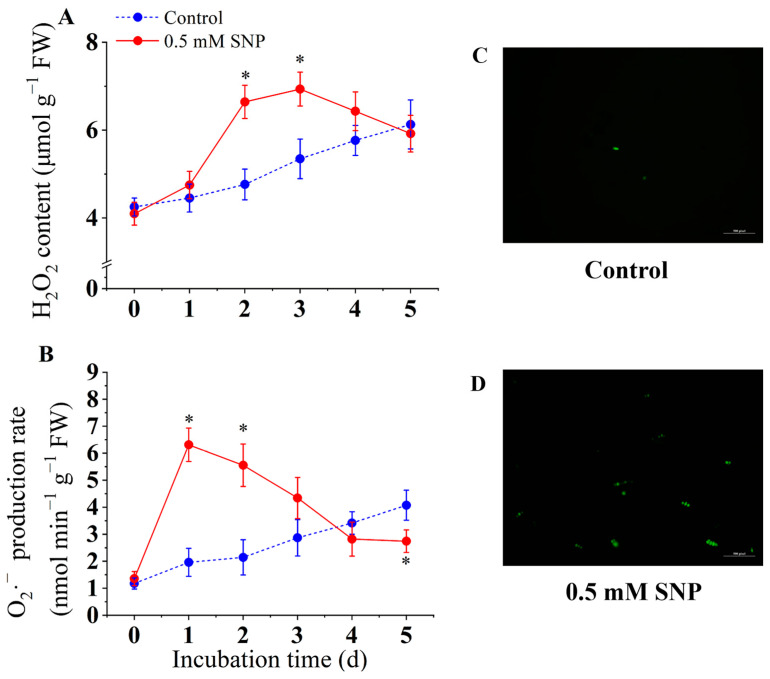
Effect of SNP on endogenous H_2_O_2_ content (**A**), production rate of O_2_·^−^ (**B**), and ROS fluorescence (**C**,**D**) of *A. alternata*. Values are presented as means ± SD (*n* = 10). The * indicates significant differences (*p* < 0.05).

According to the method of Hu et al. [8], the NO and ROS were detected by 3-amino, 4-aminomethyl-2′,7′-difluorescein diacetate (DAF-FM) and 2,7-dichlorodi-hydrofluorescein diacetate (DCHF-DA) fluorescence staining, respectively. Based on the previous study (Figure 1, Figure 2 and Figure 3), the spore suspension of *A. alternata* was added to PDB medium containing 0.5 mM SNP, and distilled water treatment was used as a control. The spores were collected by centrifugation at 12,000× *g* for 5 min after incubation at room temperature for 3 h.

The spores were then rinsed twice with phosphate buffered saline (PBS) buffer (pH 7.4), and the fluorescent probes DAF-FM and DCHF-DA were added and adjusted to concentrations of 5 μM L^−1^ and 20 μg L^−1^, respectively. Dark incubation at 37 °C for 20 min and 30 °C for 60 min was used for NO and ROS detection, respectively. The spores were then rinsed twice with PBS buffer and then observed and photographed under the fluorescence microscope (DM 2500, Leica, Heidelberg, Germany). Three replicates were used for each group.

### 2.8. The Rate of O_2_·^−^ Production and H_2_O_2_ Content Assay

The generation rate of O_2_·^−^ was assayed using a super anion activity content assay kit (BC1290, Solarbio Science and Technology, Beijing, China) according to the manufacturer’s instructions. The measured generation rate of O_2_·^−^ of samples was expressed as min^−1^ g^−1^ FW.

The H_2_O_2_ content assay was performed using a hydrogen peroxide (H_2_O_2_) assay kit (S0038–1, Beyotime Biotechnology, Shanghai, China) according to the manufacturer’s instructions. The measured H_2_O_2_ content of samples was expressed as μmol g^−1^ FW.

### 2.9. Detection of NOX, SOD, CAT, POD, APX, and GR Activities

The NOX activity assay was performed using a NADHP oxidase (NOX) assay kit (S0086, Beyotime Biotechnology, Shanghai, China) according to the manufacturer’s instructions. The measured NOX activity of samples was expressed as U mg^−1^ protein.

The SOD activity assay was performed using a superoxide dismutase (SOD) assay kit (S0086, Beyotime Biotechnology, Shanghai, China) according to the manufacturer’s instructions. The measured SOD activity of samples was expressed as U mg^−1^ protein.

The CAT activity assay was performed using a catalase (CAT) assay kit (P3541, Beyotime Biotechnology, Shanghai, China) according to the manufacturer’s instructions. The measured CAT activity of samples was expressed as U mg^−1^ protein.

The POD activity assay was performed using a peroxidase assay kit (076323, Shanghai Enzyme-linked Biotechnology Co., Ltd., Shanghai, China) according to the manufacturer’s instructions. The measured POD activity of samples was expressed as U mg^−1^ protein.

The GPX activity assay was performed using a glutathione peroxidase (GPX) activity assay kit (S0038–1, Beyotime Biotechnology, Shanghai, China) according to the manufacturer’s instructions. The measured GPX activity of samples was expressed as U mg^−1^ protein.

The GR activity assay was performed using a glutathione reductases assay kit (092942, Shanghai Enzyme-linked Biotechnology Co., Ltd., Shanghai, China) according to the manufacturer’s instructions. The measured GR activity of samples was expressed as U mg^−1^ protein.

### 2.10. Detection of Cx, β-1,3-Glucanase, PG, PMG, PGTE, and PMTE Activities In Vitro and In Vivo

#### 2.10.1. Extraction and Purification of Crude Enzyme Solution In Vitro

The assessment of CWDEs activities was based on the method of Ge et al. [25], with minor modifications. *A. alternata* was incubated on PDA medium for 7 d at 28 °C in the dark, and then agar discs (6 mm diameter) containing *A. alternata* mycelium were taken with a punch. The agar discs containing *A. alternata* mycelium were inoculated into 250 mL of PDB medium (containing 0 mM and 0.5 mM SNP, respectively) and incubated at 25 °C at 150 rpm min^−1^ with constant temperature. The mycelium was filtered, and the medium solution was collected on day 0, 1, 2, 3, 4, and 5, respectively. The medium solution was centrifuged at 12,000× *g* for 30 min at 4 °C, and the supernatant was extracted to determine the enzyme activity.

The crude enzyme solution was mixed with 60% saturated ammonium sulphate and let stand at 4 °C for 5 h. After centrifugation at 15,000× *g* for 25 min at 4 °C, the precipitate was collected and dissolved in 50 mM of acetic acid–sodium acetate buffer (pH 5.0) and then dialyzed at 4 °C for 48 h to obtain the purified enzyme solution.

#### 2.10.2. Extraction and Purification of Crude Enzyme Solution In Vivo

The *A. alternata* was cultured on potato dextrose agar (PDA) for seven days, and the spore suspension (10^6^ spores × 10^−3^ L^−1^) was prepared for inoculation. A total of 300 pear fruits were selected and randomly divided into two groups of 150 (SNP-treated group and control group), including 3 replicates of 50 each. The fruit surface was sterilized with 75% alcohol, air dried, and then uniformly punched on the surface of the tubers with a perforator (4 mm in diameter). The holes were inoculated with 20 μL of spore suspension, dried, and stored at 22 (±2 °C). Tissue samples of *A. alternata* were collected from the onset site areas of the fruit on day 0, 1, 2, 3, 4, and 5 after inoculation, respectively.

The samples (1 g) were added to 9 mL of 1 mol L^−1^ NaCl and homogenized at 0 °C. The homogenate was centrifuged at 4 °C and 12,000× *g* for 20 min, and the supernatant was collected and stored at 4 °C. The pear fruits were inoculated with spore suspensions treated with sterile water in PDB medium as a control. The difference between the activities of CWDEs in *A. alternata* inoculated with SNP-treated spores and those inoculated with sterile-water-treated spores were used to represent the activity of CWDEs secreted by *A. alternata* during infection.

#### 2.10.3. Measurement of CWDEs Activities

The Cx activity assay was performed using a cellulase assay kit (095198, Shanghai Enzyme-linked Biotechnology Co., Ltd., Shanghai, China) following the manufacturer’s instructions. The measured Cx activity of the pear fruit samples was expressed as U mg^−1^ protein.

The β-1,3-glucanase activity assay was performed using a β-1,3-glucanase assay kit (MC574L, Shanghai Enzyme-linked Biotechnology Co., Ltd., Shanghai, China) following the manufacturer’s instructions. The measured β-1,3-glucanase activity of the pear fruit samples was expressed as U mg^−1^ protein.

The PG activity assay was performed using a polygalacturonase assay kit (076398, Shanghai Enzyme-linked Biotechnology Co., Ltd., Shanghai, China) following the manufacturer’s instructions. The measured PG activity of the pear fruit samples was expressed as U mg^−1^ protein.

The PMG activity reaction system consisted of 0.5 mL of 1.0 mg mL^−1^ pectin, 1.0 mL of 50 mM L^−1^ acetate buffer (pH 5.5), and 0.5 mL of crude enzyme solution. The reaction solution was incubated at 37 °C for 1 h. After cooling, 1.0 mL of 3,5-dinitrosalicylic acid (DNS) was added, boiled for 5 min, and then rapidly cooled to room temperature. Sterile water was used instead of the crude enzyme solution as a control, and the absorbance value at 540 nm was measured after cooling to room temperature. The PMG activity was expressed as U mg^−1^ protein.

The PMTE and PGTE reaction system consisted of 1.0 mL 3.0 mmol L^−1^ CaCl_2_, 4.0 mL 50 mmol L^−1^ glycine, sodium hydroxide buffer (pH 9.0), 3.0 mL 1.0 g L^−1^ reaction substrate (with the substrates of PMTE and PGTE being pectin and polygalacturonic acid, respectively), and 0.1 mL of crude enzyme solution. The reaction system solution was incubated at 30 °C for 10 min and then cooled down, and the absorbance value at 232 nm was determined after cooling to room temperature. The crude enzyme solution was replaced with sterile water as a control. The activities of PGTE and PMTE were expressed as U mg^−1^ protein.

The total protein content was measured by using Coomasse Brilliant Blue staining [29].

### 2.11. Gene Expression Analysis by Quantitative Real-Time PCR (qRT-PCR)

The total RNA was isolated from ground tissues and the first-strand cDNA was synthesized by the cetyltrimethylammonium bromide (CTAB) method and extracted using a Takara RNA extraction kit (Takara Biotechnology, Shiga, Japan). The qRT-PCR was performed for the expression levels of *A. alternata NOS* (*AaNOS)*, *AasGC*, *AaNOXa*, *AaNOXb*, *AaSOD*, *AaCAT*, *AaAPX*, *AaGR*, *AaCx*, *Aa*β-1,3-glucanase, and *AaPG*, with primer information for amplification of the above genes given in Table S1. The SYBR Green PCR Premix Ex Taq ™ (Takara Biomedicals, Shiga, Japan), cDNA, forward and reverse primers, and ROX reference dye II were added to an ABI 7000 instrument (Applied Biosystems, Foster City, CA, USA) for reaction. The operation was as follows: at 95 °C for 10 s, at 95 °C for 5 s with 40 cycles, and at 60 °C for 40 s. The *Alternaria alternata actin (Act1) gene* (*AaActin*, MN164690.1) was used as an internal reference. The relative quantifications were then calculated using the 2^−ΔΔCT^ method, and the CT values from the *S. tuberosum* actin gene were used to normalize all the qRT-PCR reactions.

### 2.12. Statistical Analysis

Each treatment included three biological replicates, and the data were analyzed using SPSS11.0 software package (SPSS Inc., Chicago, IL, USA). The data were subjected to one-way analysis of variance (ANOVA) and Duncan‘s post hoc test, with significance set at a *p*-value < 0.05. The resulting data are presented as mean ± standard deviation. Furthermore, heat-maps were used to visualize the expression level of each gene by GraphPad Prism8.0 software (GraphPad Software, San Diego, CA, EUA).

## 3. Results

### 3.1. Effect of the SNP on the Growth of Mycelium In Vitro and In Vivo and on the Spore Germination of A. alternata

As shown in Figure 1A, low concentrations of SNP treatment did not significantly inhibit or promote the growth of *A. alternata* from the pre-growth stage until the late growth stage in vitro. There was no significant change in the colony diameter of the SNP-treated group compared to the control group (*p* < 0.05). However, when the concentration of SNP was increased to 0.75 mM, the colony diameter was significantly reduced, and the growth of *A. alternata* was inhibited (*p* < 0.05). The growth of *A. alternata* in the 0.75 mM and 1 mM SNP-treated groups was inhibited by 13.2% and 22.8%, respectively, compared to the control group (Figure 1A). The results of the in vivo experiment showed that when the concentration of SNP was increased to 1 mM L^−1^, there was significant inhibition of pear black spot disease on the damaged inoculated pears, with the diameter of pear spots in the SNP-treated group reduced by 16.03% compared to the control (Figure 1B). Below this concentration, there was no obvious effect.

The results of spore germination showed that the spore germination rate was significantly higher than that of the control group at low concentrations of SNP (*p* < 0.05). The spore germination rate of the 0.5 mM SNP-treated group was 25.9% higher than that of the control group at the fourth hour. When SNP was increased to 1 mM, *A. alternata* spore germination was inhibited, showing a slightly delayed effect (Figure 1C).

### 3.2. Effect of SNP on NOS Activity, Endogenous NO Content, and cGMP Content

The results showed that 0.5 mM SNP significantly activated NOS activity (*p* < 0.05) (Figure 2A). NOS activity in the SNP-treated group increased rapidly from day 2 of storage. Similarly, endogenous NO content increased from day 2 to day 5 (Figure 2B). In the control group, there was almost no increase in NOS activity and endogenous NO accumulation. Figure 2C shows that 0.5 mM SNP significantly accelerated cGMP accumulation during the inoculation period (*p* < 0.05). cGMP levels increased rapidly from day 2, peaking on day 3. On day 3, cGMP levels in the SNP-treated group were 2.74 times higher than in the control group.

*A. alternata* spores were stained with the DAF-FM fluorescent probe, and NO accumulation was observed by confocal microscopy (*p* < 0.05) (Figure 2D,E). The fluorescence staining results showed that 0.5 mM SNP treatment significantly promoted the production of endogenous nitric oxide in *A. alternata* spores. The fluorescence intensity of the control group was significantly lower than that of the SNP-treated group, consistent with the changes in NO content in *A. alternata*.

### 3.3. Effect of SNP on the Generation of O_2_·^−^ and H_2_O_2_ Content

As shown in Figure 3A, the H_2_O_2_ content in the SNP-treated group initially increased rapidly from day 0 to day 3, with the H_2_O_2_ content of the 0.5 mM SNP-treated group being 29.7% higher than that of the control group. The H_2_O_2_ content then gradually decreased on day 4. In the control group, the H_2_O_2_ content gradually increased from day 0 to day 5. Figure 3B shows that the O_2_·^−^ production rate in the control group increased slightly from day 0 to day 5. In the SNP treatment group, the O_2_·^−^ production rate increased sharply from day 0 to day 1 and then decreased rapidly from day 2 to day 4. On day 2, the O_2_·^−^ production rate of the 0.5 mM SNP treatment was 2.5 times higher than that of the control group. On day 5, the O_2_·^−^ production in the SNP treatment group was lower than that of the control group.

*A. alternata* spores were stained with the fluorescent probe DCFH-DA, and the accumulation of reactive oxygen species was observed by confocal microscopy (Figure 3C,D). The fluorescence staining results showed that 0.5 mM SNP treatment significantly induced the accumulation of large amounts of reactive oxygen species in *A. alternata* spores, while the fluorescence intensity of the control group was significantly lower than that of the SNP-treated group. This is consistent with the changes in H_2_O_2_ and O_2_·^−^.

### 3.4. Effect of SNP on Activities of NOX, SOD, CAT, POD, GPX, and GR

**Figure 4:**
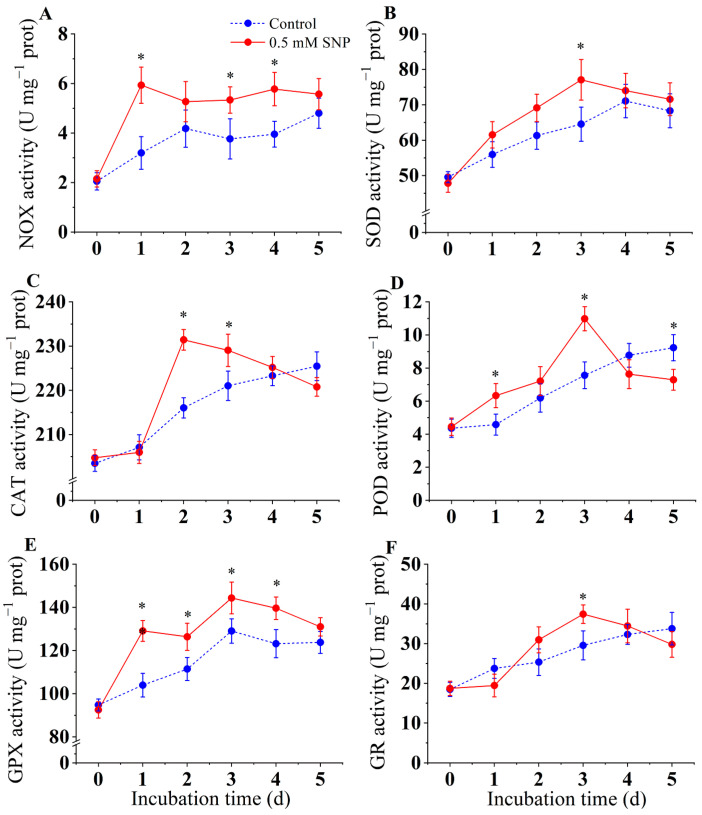
Effect of SNP on activities of NOX (**A**), SOD (**B**), CAT (**C**), POD (**D**), GPX (**E**), and GR (**F**) of *A. alternata*. Values are presented as means ± SD (*n* = 10). The * indicates significant differences (*p* < 0.05).

The results showed that SNP could significantly activate the activity of NOX compared to the control group (*p* < 0.05) (Figure 4A). NOX activity in the SNP-treated group peaked on day 3 and then decreased. On day 3, NOX activity was 48.5% higher than that of the control group.

As shown in Figure 4B, SOD activity in the SNP-treated group increased and then decreased during the inoculation period and was markedly higher than that in the control group during days 0–5 (*p* < 0.05). SOD activity in SNP-treated pears peaked on day 3, whereas the SOD activity in the control group peaked on day 4. During the inoculation period, SNP treatment effectively enhanced SOD activity in *A. alternata*. The SOD activity of the SNP-treated group was higher than that of the control group, with the greatest difference observed on day 3, when the SNP-treated group was 19.4% higher than the control group.

CAT activity in the SNP-treated group was significantly higher than that in the control group from day 1 to day 3 and lower than that in the control group at the end of the inoculation period (*p* < 0.05) (Figure 4C). The results showed that SNP enhanced the POD activity of mycelium, which increased steadily from day 0 to day 3, reaching its peak, and then decreased until day 5. In the control group, POD activity continued to increase from day 0 to day 5; however, the overall level of POD activity was lower than in the SNP-treated group (Figure 4D).

GPX activity in the SNP-treated group increased by 24.2% and 11.8% compared to the control on days 1 and 3, respectively. The highest peak of GPX activity in both treatments on day 3 was 129.11 ± 3.2 U for the control and 144.36 ± 4.8 U for the SNP treatment (Figure 4E). As shown in Figure 4F, the GR activity of the control group was generally more stable than that of the treatment group. After treatment with SNP, the GR activity of the mycelium increased significantly (*p* < 0.05), reaching a peak on day 3, which was 26.6% higher than that of the control group. Subsequently, the GR activity decreased, and the GR activity of the SNP-treated group was lower than that of the control group on day 5.

### 3.5. Effect of SNP on the Activities of CWDEs from A. alternata

#### 3.5.1. CWDEs from *A. alternata* In Vitro

**Figure 5:**
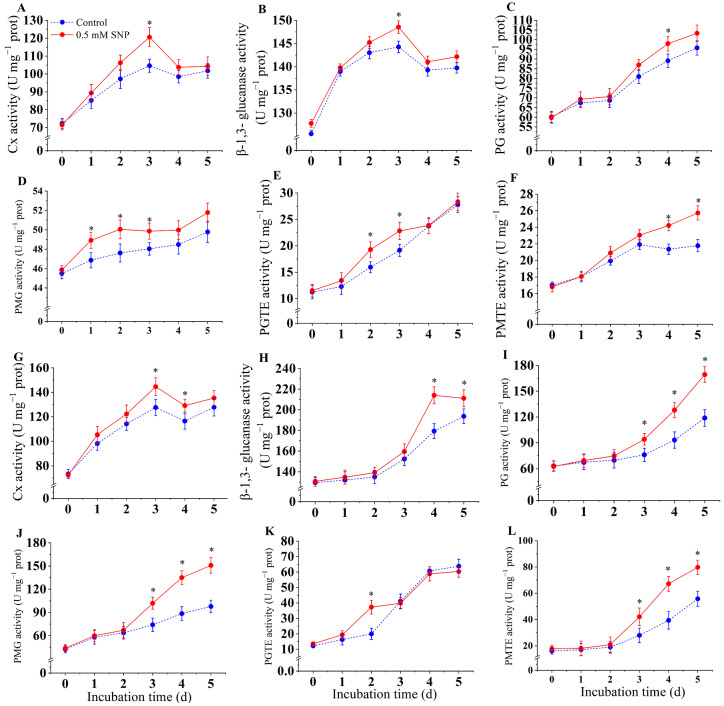
Effect of SNP on activities of Cx (**A**), β-1,3-glucanase (**B**), PG (**C**), PMG (**D**), PGTE (**E**), and PMTE (**F**) from *A. alternata* in vitro and Cx (**G**), β-1,3-glucanase (**H**), PG (**I**), PMG (**J**), PGTE (**K**), and PMTE (**L**) from *A. alternata* of pear fruit, respectively. Values are presented as means ± SD (*n* = 10). The * indicates significant differences (*p* < 0.05).

Figure 5 shows the changes in cell wall degrading enzymes of *A. alternata* in vitro. Compared to the control group, SNP treatment significantly induced an increase in Cx and β-1,3-glucanase activities (*p* < 0.05). Cx and β-1,3-glucanase activities increased from days 0 to 3 and peaked on day 3, at which time Cx activity in the SNP-treated group was 15.2% higher than that in the control group. Afterward, Cx and β-1,3-glucanase activities gradually decreased (Figure 5A,B).

PG, PMG, and PGTE activities gradually increased over time and continued to rise during the incubation period (Figure 5C–E). PG enzyme activity in the SNP-treated group increased significantly from day 0 to day 2, and PGTE enzyme activity in the SNP-treated group was significantly higher than in the control group from day 2 to day 3 (*p* < 0.05). There was no significant difference in PMG enzyme activity compared to the control (*p* < 0.05).

PMTE enzyme activity in the SNP-treated group continued to increase throughout the incubation period (Figure 5F). In the later stage of incubation (days 3–5), PMTE enzyme activity was activated by SNP treatment and was significantly higher than that of the control group (*p* < 0.05). The PMTE enzyme activity of the control group initially increased from day 0 to day 3 and gradually decreased from day 4 onwards. On the fifth day, the PMTE activity of the SNP-treated group was 18.3% higher than that of the control group (Figure 5F).

#### 3.5.2. CWDEs from *A. alternata* of Pear Fruit

Figure 5 shows the changes in cell-wall-degrading enzymes of *A. alternata* inoculated in pear fruits. Compared with the control group, SNP treatment significantly induced an increase in Cx and β-1,3-glucanase activities. These activities increased during the early inoculation period, reaching a peak on day 3 and day 4, respectively. At their peaks, the Cx and β-1,3-glucanase activities of the SNP-treated group were 13.2% and 19.3% higher than those of the control group. Afterward, the Cx activity gradually decreased while the β-1,3-glucanase activity continued to increase (Figure 5G,H).

PG, PMG, PGTE, and PMTE enzyme activities gradually increased over time and continued to rise during the incubation period (Figure 5I–L). Compared with the control group, the increase in PG, PMG, and PMTE activities was significantly induced by SNP. The PG, PMG, and PMTE activities of the SNP-treated group were 42.5%, 54.2%, and 42.9% higher, respectively, than those of the control group on day 5 after inoculation (Figure 5G,H). The activities of PMG and PGTE in the SNP-treated group were significantly higher than those of the control group on day 3 and day 2, respectively, and during the late stage of inoculation in vivo. There was no significant difference in the activities of PMG and PGTE in the SNP-treated group compared to the control group during the late stage (Figure 5K,L).

### 3.6. Effect of SNP on Relative Gene Expression Levels of A. alternata

**Figure 6:**
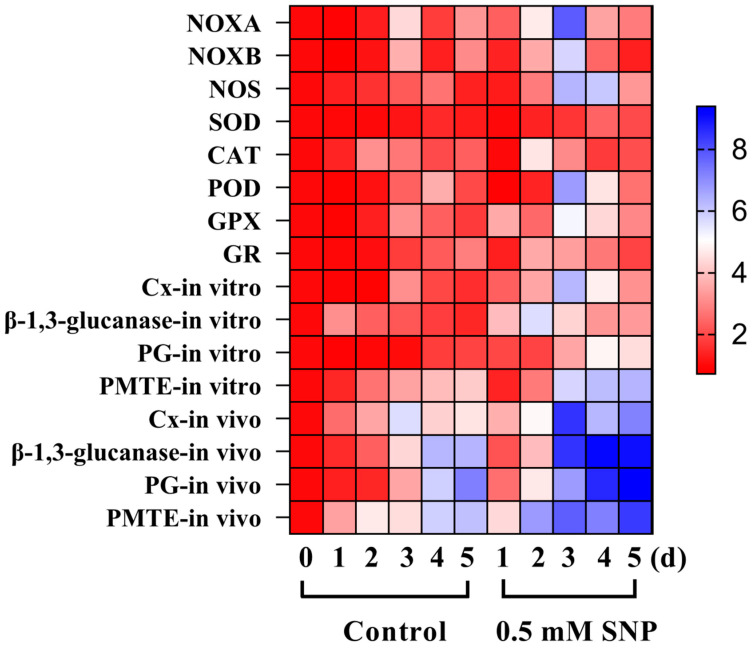
Effect of SNP on relative gene expression levels of *A. alternata*. Values are presented as means ± SD (*n* = 10).

RT-qPCR results showed that 0.5 mM SNP induced a significant upregulation in the expression of *AaNOXA*, *AaNOXB*, *AaNOS*, *AaCAT*, *AaPOD*, and *AaGPX*, with no significant change in the expression of *AaSOD* compared to the control group (*p* < 0.05) (Figure 6). On day 3, the expression levels of *AaNOXA* and *AaNOXB* were 78% and 57.2% higher in the SNP-treated group than in the control group, respectively. Meanwhile, the level of *AaNOS* gene expression in the SNP-treated group was 2.86 times higher than in the control group.

The results of in vitro experiments showed that 0.5 mM SNP significantly induced the upregulation of *AaCx*, *Aaβ-1,3-glucanase*, *AaPMG*, and *AaPMTE* expression, with no significant change in *AaPG* expression. The in vivo experiments showed that the expression levels of *AaCx*, *Aaβ-1,3-glucanase*, *AaPG*, and *AaPMTE* were significantly induced by 0.5 mM SNP, with the expression of cell-wall-degrading enzyme-related genes after inoculation in pear fruits being significantly higher than those in the in vitro experiments (*p* < 0.05). On the fifth day of inoculation, the gene expression levels of *AaCx*, *Aaβ-1,3-glucanase*, *AaPG*, and *AaPMTE* in the SNP-treated group were 57.8%, 42.3%, 29.7%, and 36.1% higher than those in the control group, respectively.

## 4. Discussion

As a common pathogen of fruits and vegetables, *A. alternata* is capable of causing black spot disease in pears, apples, apricots, peaches, and other fruits, seriously affecting fruit quality and causing significant economic losses [2]. Nitric oxide (NO) is an intracellular signaling molecule that plays a bidirectional regulatory role in both the physiological and pathological processes of pathogens [9]. In this study, we demonstrated that a low concentration of NO (0.5 mM SNP) effectively promoted mycelial growth and spore germination of *A. alternata* in vitro (Figure 1). Interestingly, while NO significantly promoted mycelial growth and spore germination in vitro, the in vivo experiments showed no significant effect of NO on colony growth or size. The results of the in vivo experiments showed that treatment with low concentrations of SNP did not significantly inhibit or accelerate mycelial growth of *A. alternata*, and there was no significant change in colony diameter in the SNP-treated group compared with the control group. Growth of *A. alternata* was inhibited when the concentration of SNP was increased to 0.75 mM (Figure 1B). The inhibitory effect of high concentrations of NO on *F. sulphureum* and *A. alternata* has been confirmed [8,30]. Meanwhile, the important function of low levels of NO in regulating spore formation and the germination of fungi has also been proven. Studies reported that low levels of NO facilitated the growth and development of *Aspergillus nidulans* and spore germination of *Puccinia striiformis*, respectively [6,31]. Low levels of NO were found to be involved in mycelial cultures of *Bambusicolous shiraia* and to promote the spore germination of *Coniothyrium minitans* by mediating the cGMP signaling pathway [32,33]. In contrast, excessive NO inhibited spore germination in *F. sulphureum* and *Botrytis cinerea* [8,34]. Our study presented similar results. The current research showed that 0.25 mM and 0.5 mM SNP significantly increased spore germination in *A. alternata* (*p* < 0.05). However, 1 mM of SNP had the opposite effect, delaying germination (Figure 1C). Therefore, we speculate that low levels of NO promote mycelial growth and spore germination of *A. alternata* by modulating a series of signaling pathways, while excessive NO exhibits cytotoxicity, affecting cellular activities and inhibiting growth and development of the pathogen [8,13,32,33]. In addition, although high concentrations of NO inhibited the growth of pathogen, it is also potentially phytotoxic to the host plant, as evidenced by the promotion of senescence and apoptosis of plant cells, which exacerbates the process of infestation by pathogen [35]. For example, excess high-level NO promoted the development of fruit diseases in grape [5] and lycium barbarum [36]. In peach fruit, high concentrations of NO treatment accelerated the development of fruit chilling injury [37]. In this study, we focused on the effects of exogenous low concentrations of NO on the growth and development of *A. alternata* and its pathogenicity. However, considering that NO treatment regulates multiple pathways in both the pathogen and the host plant during the infestation process, it is necessary to follow up to further investigate the potential mechanisms by which exogenous NO affects the host plant during the infestation process.

MDA content reflects lipid peroxidation of cell membranes, and electrolyte leakage is one of the most important indicators of cell membrane integrity [8,30]. As shown in Figure S1, low concentrations of SNP (0.25 mM and 0.5 mM) treatments decreased the MDA content and electrolyte leakage of *A. alternata*, whereas both were higher in the high-concentration SNP-treated group. Similar results were reported in our previous study on *F. sulphureum* [8]. Ergosterol, an isoprenoid derivative, is the major sterol component of fungal cell membranes and is generally present in the free state in the phospholipid bilayer, helping to maintain the stability, integrity, and fluidity of the fungal cell membrane structure. It also plays a crucial role in signal transduction, substance operation, proper localization of membrane proteins, and ensuring normal cell viability within the fungal cell [38]. It has been demonstrated that ergosterol reduction causes changes in sterol fractions, which disrupt the cellular structure and affect the operation of the normal function of the plasma membrane of fungal cells [38]. Ergosterol depletion causes damage to the cell structure, especially cell membrane integrity, which is accompanied by increased MDA content and electrolyte leakage, as confirmed in *Penicillium digitatum* and *Penicillium expansum* [38,39]. Figure S1 showed that treatment with 0.25 mM SNP significantly slowed down the decrease in ergosterol content in *A. alternata*, whereas treatment with high concentrations of SNP (0.75 mM and 1 mM) inhibited its synthesis, which is consistent with the changes in MDA content and electrolyte leakage. These results may be attributed to the fact that low concentrations of NO exert a positive effect on the physiological processes of *A. alternata*. At the same time, as a free radical gas molecule, high levels of NO can cause cell damage and disrupt the cellular structure of *A. alternata* [38,39].

In fungi, NO is mainly catalyzed by NOS-like enzymes in the cytoplasm, generating NO from arginine [9]. NO activates the synthesis of cGMP, which is a second messenger molecule involved in the regulation of multiple signaling pathways in fungi, affecting growth and development, particularly the formation and germination of fungal spores [32]. The previous studies have reported NOS-like catalyzed the conversion of arginine to citrulline in *Aspergillus nidulans* and *B. cinerea*, accompanied by NO generation [40,41]. The current study showed that 0.5 mM SNP significantly induced an increase in NOS activity accompanied by a significant increase in endogenous NO levels in *A. alternata* (Figure 3). Additionally, as expected, the 0.5 mM SNP treatment increased cGMP levels (Figure 3), consistent with the spore germination results. In *Blastocladiella* and *Schizosaccharomyces* pombe, NO was found to regulate spore germination by mediating the cGMP signaling pathway, and spore germination was inhibited when the NO-cGMP signaling pathway was blocked [42,43]. A similar conclusion was reached in the present study, where low concentrations of exogenous NO might induce an increase in NOS activity and endogenous NO accumulation in *A. alternata*, which, in turn, activated cGMP synthesis and thus accelerated spore germination.

Fungal infection induces massive production and accumulation of reactive oxygen species (ROS) in plants, which is also considered to be one of the earliest responses of host plants to pathogen invasion. Some studies have observed early ROS production in the host during infection of various plants, such as *Arabidopsis thaliana* [44], tomato [45], and potato [8], which is considered to be one of the host resistance responses [46]. However, it is interesting to note that ROS play an important role in plant infection by pathogens [2]. *A. alternata* facilitates the infection process by forming infection structures and synthesizing ROS to destroy host plant tissues [2]. The host plant generates large amounts of ROS when attacked by pathogens to fight against the pathogen, and the pathogen responds to the oxidative stress by activating antioxidants [2]. The results of this study showed that the O_2_·^−^ content in *A. alternata* peaked after one day of 0.5 mM SNP treatment and decreased thereafter. On the other hand, the H_2_O_2_ content increased sharply on the second day after SNP treatment, peaked on the third day, and gradually decreased thereafter. O_2_·^−^ was observed to be rapidly induced at an early stage in this study, which may be one of the reasons for the proliferation of pathogen (Figure 3). Furthermore, in view of the simultaneous observation of an increase in SOD enzyme activity induced by SNP in *A. alternata* (Figure 4), the elevated H_2_O_2_ content in *A. alternata* can be attributed, on the one hand, to the inducing effect of SNP and, on the other hand, to the conversion of O_2_·^−^ to H_2_O_2_ by SOD [14]. Similar studies have shown that low concentrations of NO can induce ROS generation at an early stage of infection to facilitate infection in rice blast fungus and *Aspergillus flavus* [12,47].

NOX plays an important role in the accumulation of ROS in pathogens by catalyzing oxygen molecules to O_2_·^−^. Genes encoding NOX-family enzymes have been found in fungi and associated with a wide range of functions in growth and development, physiological processes, and pathogenicity. In *Penicillium expansum*, a NOXA knockout mutant negatively regulated growth and development [20]. FgNOXD was identified in *Fusarium graminearum*, where the NOXD deletion mutant showed attenuated growth and conidia formation, while sexual development was completely abolished [48]. Studies have shown that NOX mediates ROS generation and regulates appressorium formation in *Verticillium dahliae* [49]. In the current experiment, the NOX activity was activated by low levels of NO in *A. alternata* (Figure 4A). Accordingly, the expression levels of *AaNOXA* and *AaNOXB* also increased at an early stage (Figure 6), consistent with the changes in NOX activity. The theory that NOX activity is positively correlated with ROS accumulation has been demonstrated in *p. expansum* [20], *V. dahliae* [49], and *F. sulphureum* [8]. In our present study, exogenous NO significantly increased NOX activity and cellular ROS levels in *A. alternata*, and the corresponding gene transcript levels also increased. As expected, the earlier ROS burst and subsequently enhanced pathogenicity in *A. alternata* were attributed to the effect of exogenous NO.

Excessive ROS accumulated in plants during the infection stage caused by the pathogen leads to oxidative damage to the pathogen, thus delaying the infection process [2]. The ROS scavenging system in fungi (including antioxidant enzymes such as SOD, CAT, POD, GPX, and GR) reduces the toxicity of ROS, maintains intracellular oxidative homeostasis, and ultimately promotes infection [14]. Superoxide anion (O_2_·^−^) is generated in the mitochondria and converted to H_2_O_2_ and O_2_ by the action of SOD. CAT and POD further convert H_2_O_2_ to H_2_O and O_2_ [14]. GPX and GR are jointly involved in the ASA-GSH cycling pathway. GR catalyzes oxidized glutathione (GSSG) to GSH, which facilitates ASA regeneration. GPX catalyzes reduced glutathione (GSH) and oxidizes it to oxidized glutathione (GSSG), converting H_2_O_2_ to water, thereby reducing intracellular oxidative damage [14]. The previous studies have shown similar findings linking the activation of antioxidant enzymes to the alleviation of oxidative stress in fungi during the infection process. During infection in *A. alternata*, SOD, CAT, and GPX activities were increased in response to the ROS burst [21]. In our experiment, a lot of ROS were induced by SNP in the early period of inoculation in *A. alternata*. Meanwhile, in the late period of inoculation, low levels of NO induced increased SOD and CAT activities in *A. alternata*, while activating the ASA-GSH pathway, causing an increase in GPX and GR activities and facilitating the scavenging of O_2_·^−^ and H_2_O_2_ [2]. SNP treatment resulted in a rapid decrease in SOD, CAT, and POD activities after reaching a peak, which might be related to the enhanced ROS scavenging capacity [14]. The mechanism is related to the upregulation of the expression of genes related to oxidation-reduction reactions at the transcriptional level [2,14]. It has been reported that exogenous NO increased the activities of SOD, CAT, and GPX in *Agaricus bisporus* and reduced the accumulation of hydrogen peroxide to protect organisms from oxidative stress [50]. In *Pisolithus* sp., NO inhibited intracellular ROS levels by enhancing CAT activity, promoting ASA-GSH recycling, and thus reducing cellular oxidative damage [51]. These findings also provided previous evidence for the thoughts presented in the present study.

In plant tissues, most of the cell wall components are polysaccharides, and cell-wall-degrading enzymes produced by the pathogen during the infection process are able to degrade the polysaccharides of the plant cell wall, which, in turn, helps the pathogen to infect the host plant [26]. For example, cell-wall-degrading enzymes generated by *A. alternata* help to infect plants and cause brown spot disease in citrus [52]. PG and Cx from *Penicillium digitatum* promote the infection of postharvest citrus fruit [53]. The present results indicate that low levels of NO induced an increase in the cell-wall-degrading enzyme activities of *A. alternata* in vitro and in vivo (in pear fruits inoculated with *A. alternata*), respectively. In contrast to the results of the mycelial growth and spore germination experiments, the effect of NO on cell wall-degrading enzymes may be even more remarkable in vivo. In the in vivo assay, PG activity of *A. alternata* increased in the SNP-treated group, reaching a maximum on day 2, and then continued to decrease with storage time, whereas cellulase activity continued to increase from day 3 onwards. Ramos et al. [54] reported that PG was first activated by infecting soybean with *Colletotrichum truncatum*, which assisted PMG and Cx in degrading the cell wall components of soybean. Our experiments showed similar results. Therefore, it is cautiously speculated that *A. alternata* first secretes PG to degrade the fruit cell wall in vivo, and the degraded cell wall, while providing a carbon source for *A. alternata*, also facilitates cellulase degradation of other components in the fruit cell wall, ultimately accelerating the infection. Moreover, the significant increase in *A. alternata* cell-wall-degrading enzymes in vivo might be attributed to the combination of intracellular NO signaling and the response to the plant defense of *A. alternata* [40].

The above results indicated that lower concentrations of exogenous NO improved the pathogenicity of *A. alternata* by stimulating endogenous NO and cGMP, regulating ROS metabolism, and activating cell-wall-degrading enzymes. Therefore, we conservatively hypothesize that NO might be involved in cGMP generation and ROS metabolism and that these signaling molecules are involved in the infection process. Additionally, whether NO signaling is involved in other infection-related signaling pathways needs further investigation.

## 5. Conclusions

In conclusion, our present study indicated that a lower concentration of NO (0.5 mM SNP) treatment could promote the pathogenicity of *A. alternata*. The growth of *A. alternata* was accelerated, and its pathogenicity was enhanced by increasing endogenous NO levels, ROS levels, and the activities and gene expression of cell-wall-degrading enzymes. Additionally, the activated antioxidant system, including SOD, CAT, POD, GR, and GPX, supported redox balance during the infection, which ultimately contributed to the promotion of *A. alternata* pathogenicity. However, excessive concentrations (0.75 and 1 mM SNP) showed the opposite effect on pathogenicity, likely due to structural damage to *A. alternata*. Given that pathogenicity is likely regulated by other potential factors, further studies at the molecular level are needed for a better understanding.

## Data Availability

The raw data supporting the conclusions of this article will be made available by the authors on request.

## Supplementary Materials

The following supporting information can be downloaded at: https://www.mdpi.com/article/10.3390/jof10100726/s1, Figure S1: Effect of SNP on electrolyte leakage (A), MDA content (B), and ergosterol content (C) of *A. alternata*. Values are presented as means ± SD (*n* = 10). The * indicates significant differences (*p* < 0.05); Table S1: Primer sequences used for real-time quantitative PCR (qRT-PCR).
