# Supplementary material for: Exogenous Nitric Oxide Induces Pathogenicity of Alternaria alternata on Huangguan Pear Fruit by Regulating Reactive Oxygen Species Metabolism and Cell Wall Modification
Source: J Fungi (Basel). 2024 Oct 19;10(10):726. doi: 10.3390/jof10100726 (PMC11508668; doi:10.3390/jof10100726)
Supplement: Supplementary file 1 [file jof-10-00726-s001.zip › jof-3241608-supplementary.pdf]

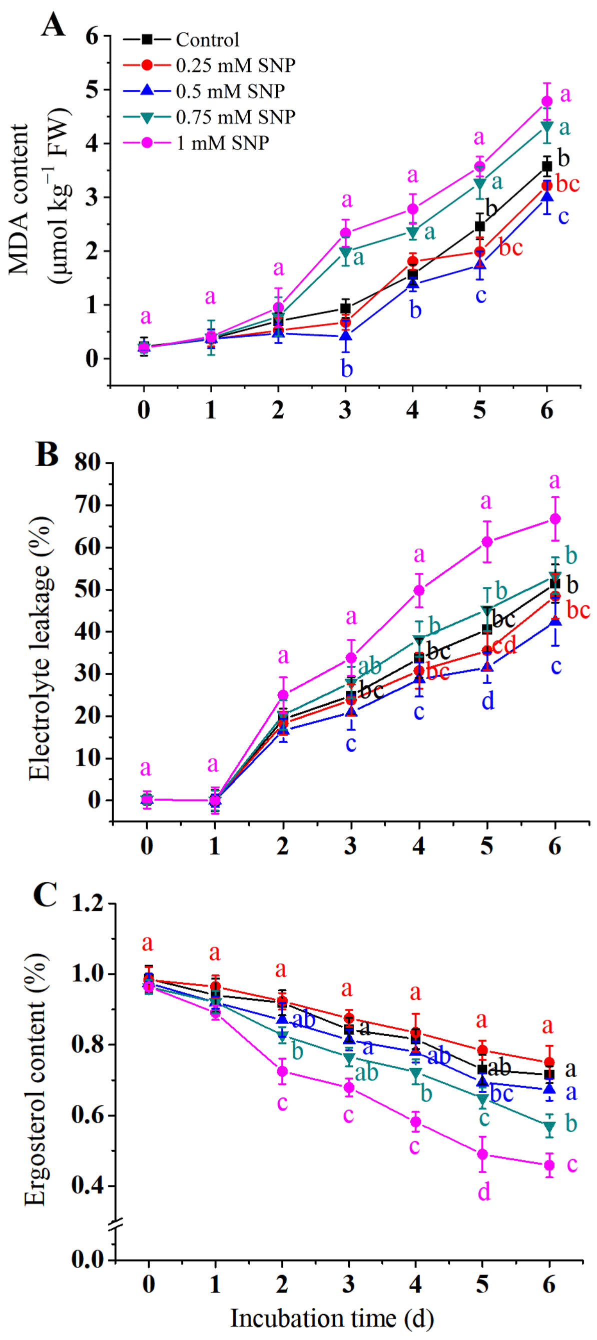

Figure S1. Effect of SNP on electrolyte leakage (A), MDA content (B), and ergosterol content (C) of *A. alternata*. Values are presented as means ± SD (n = 10). The * indicates significant differences (p < 0.05).

Table S1. Primer sequences used for real-time quantitative PCR (qRT-PCR)

Gene	Forward	Reverse	product size (bp)
Actin	GCCCCGAGCAGTCTTCCCTTC	ACGAGTCCTTCTGGCCCATACC	80
NOXA	GGACCCACTCACCGAACTCAAATC	CCATCTCGCATACCGCAGAACAG	81
NOXB	GTGCTGCCCTGAAATCTCCATCTG	CTTCCTCTCCGTGCTACAACCAAG	148
SOD	GTCAAGGCTGTCGCTGTCGTC	GTCAAGGCTGTCGCTGTCGTC	112
CAT	CGCTCCTCGTTCCGTAAGATTCAG	CGCTCCTCGTTCCGTAAGATTCAG	114
APX	ACAAGTTCAACCAGAGAAGTTCGTG	AATAGTTCGTCGGGAGAGGCTTATC	144
GR	CCTCCTCCTCGGCTGCTGAC	CACCGCTACCACCACCAATGAC	87
Cx	CACCTCGCTCGCTCCTTTCC	CCATATCCAGCAGGCTCAACATTG	132
PMTE	CAGAAGTGGAACGGTGACAACAAC	TGATAGGCACAGGCTTCGCAAG	127
PG	CCGCTGGTGGGATGGAGAGG	AGGTTGTCCGACTGAATGCTGAAG	144
